# Identification of key genes of papillary thyroid carcinoma by integrated bioinformatics analysis

**DOI:** 10.1042/BSR20201555

**Published:** 2020-08-17

**Authors:** Gang Xue, Xu Lin, Jing-Fang Wu, Da Pei, Dong-Mei Wang, Jing Zhang, Wen-Jing Zhang

**Affiliations:** 1 Department of Otorhinolaryngology Head and Neck Surgery, The First Affiliated Hospital of Hebei North University, Zhangjiakou 075000, China; 2 Department of Histology and Embryology, Hebei North University, Zhangjiakou 075000, China

**Keywords:** bioinformatics, key gene, papillary thyroid carcinoma, RNA-Seq

## Abstract

Background: Papillary thyroid carcinoma (PTC) is one of the fastest-growing malignant tumor types of thyroid cancer. Therefore, identifying the interaction of genes in PTC is crucial for elucidating its pathogenesis and finding more specific molecular biomarkers.

Methods: Four pairs of PTC tissues and adjacent tissues were sequenced using RNA-Seq, and 3745 differentially expressed genes were screened (*P*<0.05, |logFC|>1). The enrichment analysis indicated that the vast majority of differentially expressed genes (DEGs) may play a positive role in the development of cancer. Then, the significant modules were analyzed using Cytoscape software in the protein–protein interaction network. Survival analysis, TNM analysis, and immune infiltration analysis of key genes were analyzed. And the expression of ADORA1, APOE, and LPAR5 genes were verified by qPCR in PTC compared with matching adjacent tissues.

Results: Twenty-five genes were identified as hub genes with nodes greater than 10. The expression of 25 genes were verified by the GEPIA database, and the overall survival and disease-free survival analyses were conducted with Kaplan–Meier plotter. We found only three genes were confirmed with our validation and were statistically significant in PTC, namely ADORA1, APOE, and LPAR5. Further analysis found that the mRNA levels and methylation degree of these three genes were significantly correlated with the TNM staging of PTC. And these three genes were related to PTC immune infiltration. Verification of the expression of these three genes by RT-qPCR and Western blot further confirmed the reliability of our results.

Conclusion: Our study identified three genes that may play key regulatory roles in the development, metastasis, and immune infiltration of papillary thyroid carcinoma.

## Introduction

Thyroid carcinoma is presently the malignancy with the most rapidly increasing incidence in the world and is the most widely recognized endocrine carcinoma in the Western world [1]. Thyroid cancers, derived from follicular thyroid cells, can be sorted into papillary thyroid carcinoma (PTC), follicular thyroid carcinoma (FTC), and anaplastic thyroid carcinoma (ATC) according to the histological subtype [2]. Clinical results vary across these subtypes.

The annual rate of thyroid cancer has more than doubled within the past two decades, with the vast majority of this increase being ascribed to PTC, which accounts for 80–85% of all thyroid carcinomas [1]. In addition, patients with PTC suffer from cervical lymph nodes metastasis or remote metastasis, which leads to unfavorable results, and approximately 10–15% of cases may progress to a potentially fatal recurrent ailment [3,4]. Due to these reasons, uncovering the causes of PTC and its fundamental mechanisms and finding molecular biomarkers for early diagnosis and customized treatment are significant and important tasks.

With the advancement and continuous improvement of gene sequencing and gene-editing technology, it is now convenient to recognize the hub biomarkers related to neoplasm metastasis and survival status using a large amount of information available by applying bioinformatics [5]. Currently, there are no effective sensitive biomarkers for early diagnosis, treatment, and prevention of lymph node metastasis of PTC. An examination of differentially expressed genes (DEGs) between tumor and paracarcinoma tissue may help identify critical biomarkers of papillary thyroid carcinoma. As a form of molecular marker, mRNA, containing the most abundant genetic information, is necessary for protein translation, and it is separate from the pathological process of cancer at various stages [6]. Some studies utilized public databases such as The Cancer Genome Atlas (TCGA) and the Gene Expression Omnibus (GEO) to identify significant biomarkers of papillary thyroid carcinoma. However, these investigations were only founded on single datasets with constrained sample sizes or just based on online databases used to screen out the DEGs.

In the present study, we analyzed the DEGs in PTC tissues versus the matched adjacent tissues by RNA-Seq and bioinformatics methods to obtain the DEGs. Then, we screened out the key modules and extracted the key genes in those modules by constructing DEGs interaction network. Then, the possible role of differentially expressed genes was analyzed using GO annotation and KEGG pathway enrichment analysis. The expression validation, survival analysis, and functional enrichment analysis of key genes were conducted by using relevant databases. Finally, we found that the three genes ADORA1, APOE, and LPAR5 were highly expressed in PTC and were associated with PTC methylation, TNM staging, and immune infiltration.

## Methods

### Tissue samples

Thirty pairs of PTC and adjacent tissues were collected from 2019, January to 2019, July at the First Affiliated Hospital of Hebei North University. This experiment was approved by the Ethical Committee of the First Affiliated Hospital and all patients provided informed consent. All tissues were frozen in liquid nitrogen after surgical resection.

### RNA library construction and sequencing

Total RNA was isolated from four adjacent normal and cancerous thyroid samples utilizing TRIzol reagent (Qiagen, Valencia, CA, U.S.A.) as indicated by the manufacturer’s guidelines. RNAs of PTC tissues and paracancerous tissues (sample numbers: 1C, 1P, 2C, 2P, 3C, 3P, 4C, 4P; the number represents different samples, the “C” indicates a cancer sample, and the “P” represents a matched paracancerous tissue sample) were used. Six libraries were built utilizing an Illumina standard kit as indicated by the manufacturer's protocol. All sequencing was carried out on an Illumina Hiseq 4000 (LC Bio, China).

### Differentially expressed genes screening

The level of expression of mRNAs was evaluated using StringTie by calculating FPKM [7]. The DEGs between PTC and paracancerous tissue were screened with | log_2_ (fold change)|>1, and *P*<0.05 was regarded as statistically significant; the analyses were conducted using the R package Ballgown [8].

### Functional enrichment analysis and pathway analysis

To reveal the functional roles of the DEGs, the Annotation, Visualization and Integrated Discovery function annotation tool (DAVID, http://david.abcc.ncifcrf.gov/home.jsp) was used to perform Gene Ontology (GO) enrichment analysis and Kyoto Encyclopedia of Genes and Genomes (KEGG) pathway enrichment analysis. *P* values less than 0.05 were considered as cutoff criteria.

### PPI network construction and identification of hub genes

PPI networks were constructed successively using STRING database (http://string-db.org) [9]. The interactions of DEGs with a combined score > 0.7 were set as significant and Cytoscape software (version 3.7.2) was utilized to visualize and analyze the results of the STRING database. To find key (hub) genes in this PPI network, the significant module was analyzed by using the plug-in MCODE of Cytoscape software. The criteria for selection were set to the default. The key genes were chosen with degrees ≥10. Subsequently, genes in that module were used to analyse their functional roles with FunRich software.

### Data validation and analysis

To verify the accuracy of our RNA-seq results, we used the Gene Expression Profiling Interactive Analysis database to verify the expression of 25 key genes in PTC and adjacent tissues. The overall survival and disease-free survival analyses were performed by Kaplan–Meier plots for these PTC-related hub genes. Genetic alterations of 25 hub genes in PTC and their correlations with other genes were analyzed utilizing the cBioPortal for Cancer Genomics. Hub genes related to clinicopathological features were analyzed using the online database UALCAN (http://ualcan.path.uab.edu) [10]. The correlation of ADORA1, APOE, and LPAR5 expression with the immune infiltration level in PTC and the expression of these three genes in different kinds of cancers was performed using the Tumor Immune Estimation Resource database [11].

For qRT-PCR analysis, total RNA was isolated from 30 normal and cancerous papillary thyroid samples utilizing TRIzol reagent (Qiagen, Valencia, CA, U.S.A.). cDNA was synthesized with RNA reverse transcription kit (TIANGEN BIOTECH., Beijing, China). qRT-PCR was performed with an ABI 7300 Real-Time PCR System (Applied Biosystems Life Technologies, U.S.A.). The expression of the genes of interest was normalized to β-actin. The primers for ADORA1, APOE, LPAR5, and β-actin are shown in Table 1.

**Table 1:** PCR primers

Gene symbol	Primer sequence
ADORA1	F:5′-CCACAGACCTACTTCCACACC-3′
	R:5′-TACCGGAGAGGGATCTTGACC-3′
β-Actin	F:5′-CACTCTTCCAGCCTTCCTTCC-3′
	R:5′-AGGTCTTTGCGGATGTCCAC-3′
APOE	F:5′- GTTGCTGGTCACATTCCTGG -3′
	R:5′- GCAGGTAATCCCAAAAGCGAC′
LPAR5	F:5′- CACTTGGTGGTCTACAGCTTG-3′
	R:5′- GCGTAGTAGGAGAGACGAACG-3′

bp, base pair; F, forward primer; R, reverse primer.

For Western blot, RIPA buffer was used to extract protein from four pairs of tissue from PTC patients and the protein concentrations were measured via BCA methods. Briefly, the SDS-PAGE gel was used for electrophoresis and PDVF membrane was used for transmembrane transfer. PDVF membrane was blocked and then incubated with primary anti-ADORA1 antibodies (1:500 dilution, Bioss, bs-6649R), APOE (1:1500 dilution, Bioss, bs-4892R), LPAR5 antibodies (1:200 dilution, Bioss, bs-15366R), and β-actin (1:5000 dilution, Bioss, bs-0061R) at 4°C overnight followed by incubation with secondary antibodies (Zhongshanjinqiao) (1:1000 dilution) at 37°C for 1 h. The signal was detected using ECL method.

### Statistical analysis

All the data were analyzed by R and SPSS 17.0 (SPSS Inc, U.S.A.). Kaplan–Meier method was used to estimate the significant difference in survival between the over-expression group and the low-expression group of key genes in papillary thyroid carcinoma. The statistical difference was set at *P* <0.05

## Results

### Differentially expressed genes screening based on RNA-Seq

**Figure 1:**
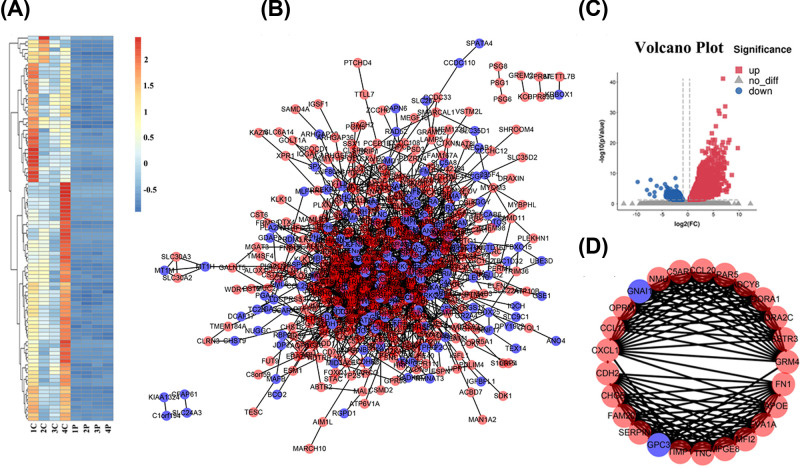
Identification of DEGs by RNA-seq The heat map (**A**) and PPI network of the DEGs (**B**). (**C**) The volcano plots of the DEGs. (**D**) The most significant module was selected by MCODE in Cytoscape. Red represents the up-regulated genes, and blue represents the down-regulated genes.

To screen out the genes or modules that may play a role in promoting cancer in papillary thyroid carcinoma, we performed RNA-Seq experiments on four pairs of thyroid cancer tissues and their matched paracancerous tissues to obtain differentially expressed genes. After RNA-Seq, we acquire 9–11 million reads for each sample. The fold changes between PTC cancer tissues and matched paracancerous samples were calculated. Setting the cut-off criterion as *P* value <0.05 and a fold change >1, there were 1927 up-regulated and 1818 down-regulated genes. These 3745 DEGs were considered to be candidate genes for subsequent study. Figure 1A showed the expression of the top 80 genes in PTC versus matched paracancerous tissues.

### Functional enrichment analysis and pathway analysis

**Figure 2:**
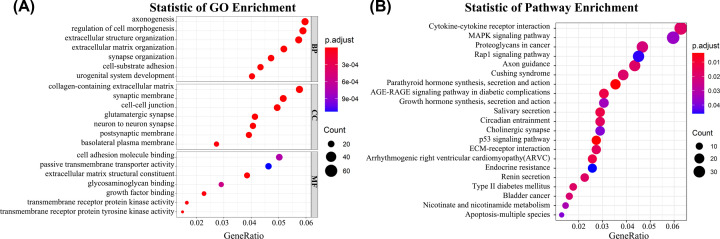
GO and KEGG pathway enrichment analysis of 3745 DEGs through RNA-Seq (**A**) Bubble plot of Gene Ontology enrichment analysis of DEGs. (**B**) Bubble plot of Kyoto Encyclopaedia of Genes and Genomes pathway enrichment analysis of DEGs.

Considering that there were many false-positive genes among these 3745 DEGs, we verified our results one by one through the TCGA database. We found that only 2462 genes in our data were consistent with the gene expression of the TCGA database. To investigate the potential function of these DEGs in PTC, genes functional enrichment was conducted by using GO and KEGG pathway analyses. For the biological process category, the DEGs were significantly involved in the regulation of axonogenesis, regulation of cell morphogenesis, extracellular structure organization, extracellular matrix organization, synapse organization, cell-substrate adhesion, and urogenital system development. The cellular component category results showed PTC-related DEGs were enriched in collagen-containing extracellular matrix, synaptic membrane, cell–cell junction, glutamatergic synapse, neuron-to-neuron synapse, postsynaptic membrane, basolateral plasma membrane. DEGs in molecular function were mainly involved in cell adhesion molecule binding, passive transmembrane transporter activity, extracellular matrix structural constituent, glycosaminoglycan binding, growth factor binding, transmembrane receptor protein kinase activity, and transmembrane receptor protein tyrosine kinase activity (Figure 2A).

As Figure 2B showed, the KEGG pathway results showed DEGs were enriched in cytokine–cytokine receptor interaction, MAPK signaling pathway, proteoglycans in cancer, Rap1 signaling pathway, axon guidance, Cushing syndrome, parathyroid hormone synthesis, secretion and action, AGE-RAGE signaling pathway in diabetic complications, growth hormone synthesis, secretion and action, salivary secretion, circadian entrainment, cholinergic synapse, p53 signaling pathway, ECM–receptor interaction, arrhythmogenic right ventricular cardiomyopathy (ARVC), endocrine resistance, renin secretion, Type II diabetes mellitus, bladder cancer, nicotinate and nicotinamide metabolism, and apoptosis-multiple species.

### PPI network construction and module analysis

**Figure 3:**
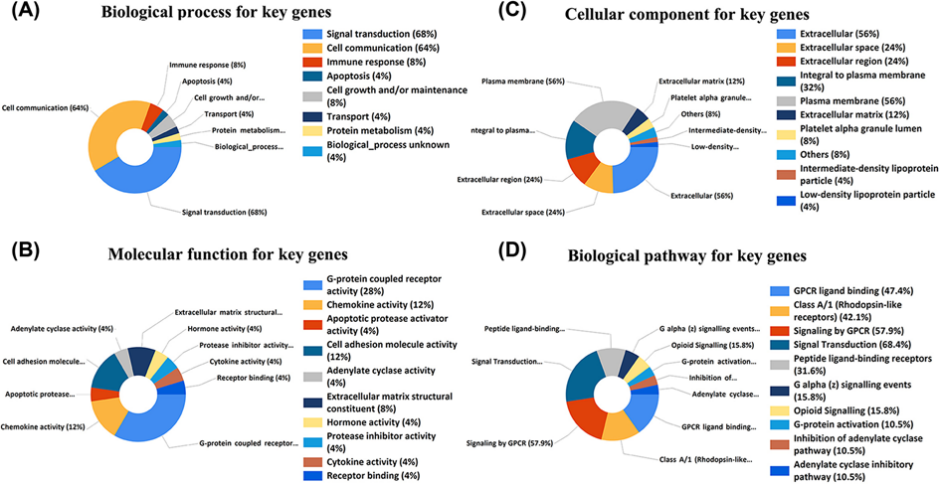
GO enrichment analysis and KEGG analysis for the key genes (**A**) Top 10 elements involved in biological processes. (**B**) Top 10 elements involved in molecular function. (**C**) Top 10 elements involved in cellular components. (**D**) Top 10 pathways related to the 25 key genes through KEGG analysis.

PPI networks were constructed successively by the database by loading the PTC related DGEs into the STRING database (Figure 1B,C). Using Cytoscape, we analyzed the most significant module in the PPI network (Figure 1D). The PPI network consisted of 587 nodes and 1836 edges. Following the use of MCODE in Cytoscape, the significant module was selected. The 25 top hub genes ADCY8, ADORA1, ADRA2C, APOE, C5AR1, CCL13, CCL20, CDH2, CHGB, CXCL12, EVA1A, FAM20A, FN1, GNAI1, GPC3, GRM4, LPAR5, MELTF or MFI2), MFGE8, NMU, OPRM1, SERPINA1, SSTR3, TIMP1, and TNC were evaluated by degree (>10) in the PPI network (Figure 1D). The results showed that the functions of the 25 key genes were mainly concentrated in signal transduction, cell communication, G-protein coupled receptor activity, cell adhesion molecule activity, and GPCR ligand binding (Figure 3).

### Data analysis and validation

**Figure 4:**
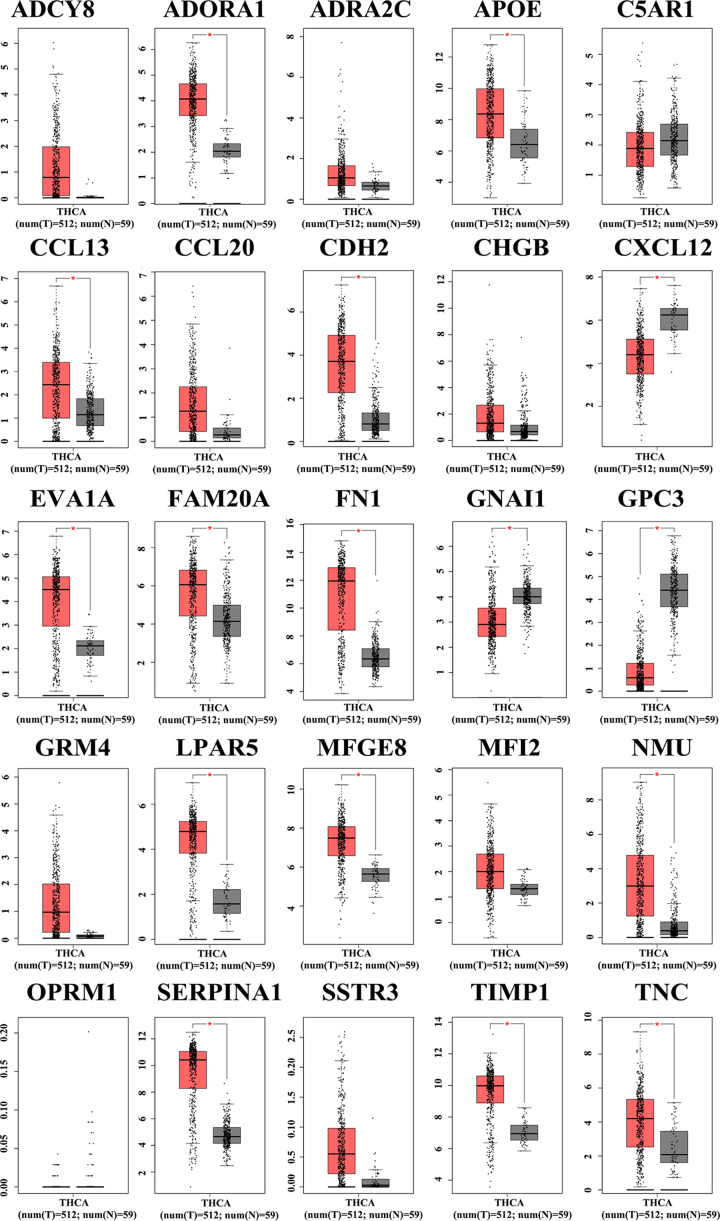
Validation of the 25 key DEGs in the GEPIA database ADORA1, APOE, CCL13, CDH2, CXCL12, EVA1A, FAM20A, FN1, GNAI1, LPAR5, MFGE8, NMU, SERPINA1, TIMP1, and TNC are overexpressed in PTC tissues compared with paracancerous tissue, while GNAI and GPC3 are down-regulated.

**Figure 5:**
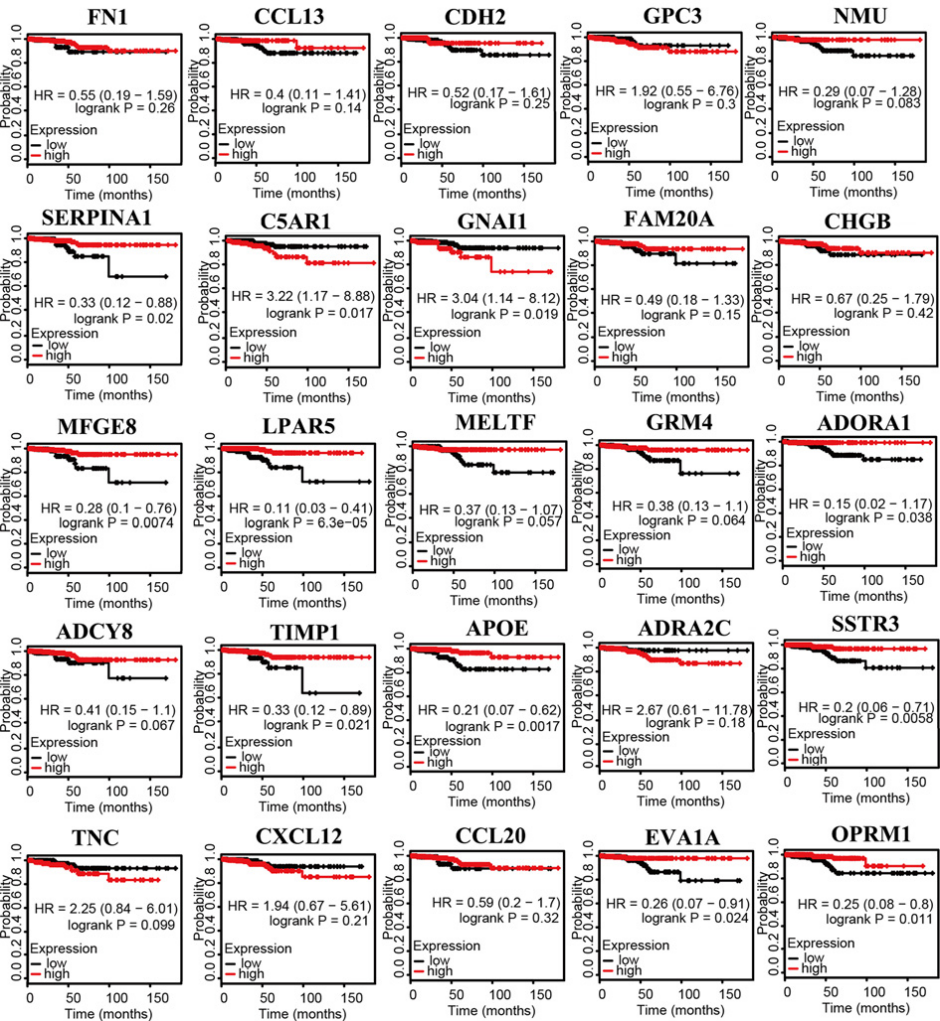
Overall survival analysis of 25 key genes in PTC using Kaplan–Meier plots Expression levels of ADORA1, APOE, C5AR1, EVA1A, FAM20A, GNAI1, LPAR5, MFGE8, OPRM1, SERPINA1, SSTR3, and TIMP1 are related to the overall survival of patients with PTC.

**Figure 6:**
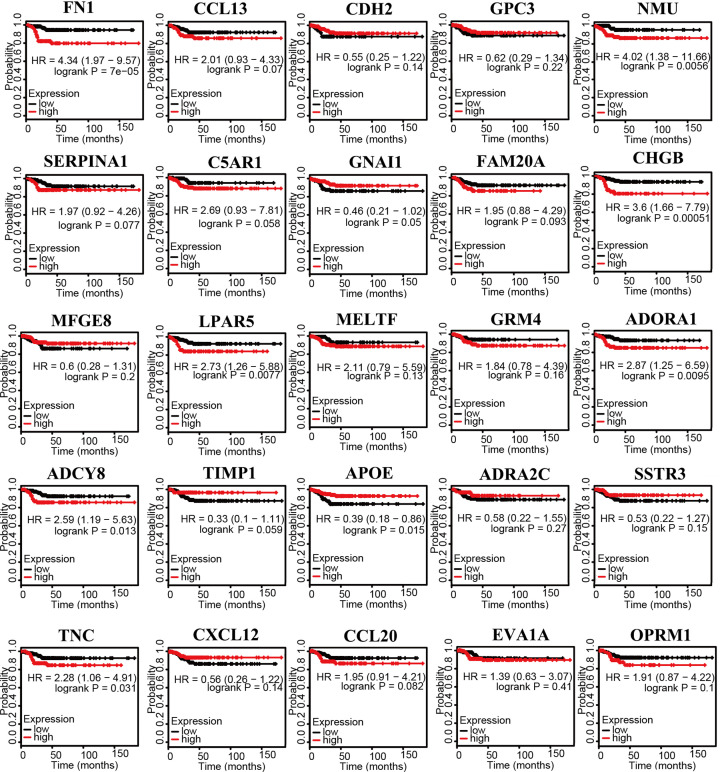
Disease-free survival analysis of 25 key genes in PTC using Kaplan–Meier plots Expression levels of ADCY8, ADORA1, APOE, CHGB, FN1, LPAR5, NMU, and TNC are significantly related to the disease-free survival of patients with PTC.

After the key genes were selected, the expression of 25 key genes in PTC and its adjacent tissues were verified by the GEPIA database (Figure 4). ADORA1, APOE, EVA1A, LPAR5, MFGE8, OPRM1, SERPINA1, SSTR3, and TIMP1 were positively related to the overall survival analysis of PTC patients, while C5AR1 and GNAI1 were negatively related (Figure 5). ADCY8, ADORA1, CHGB, FN1, LPAR5, NMU, and TNC showed positive associations with disease-free survival analysis of PTC patients but not APOE (Figure 6).

**Figure 7:**
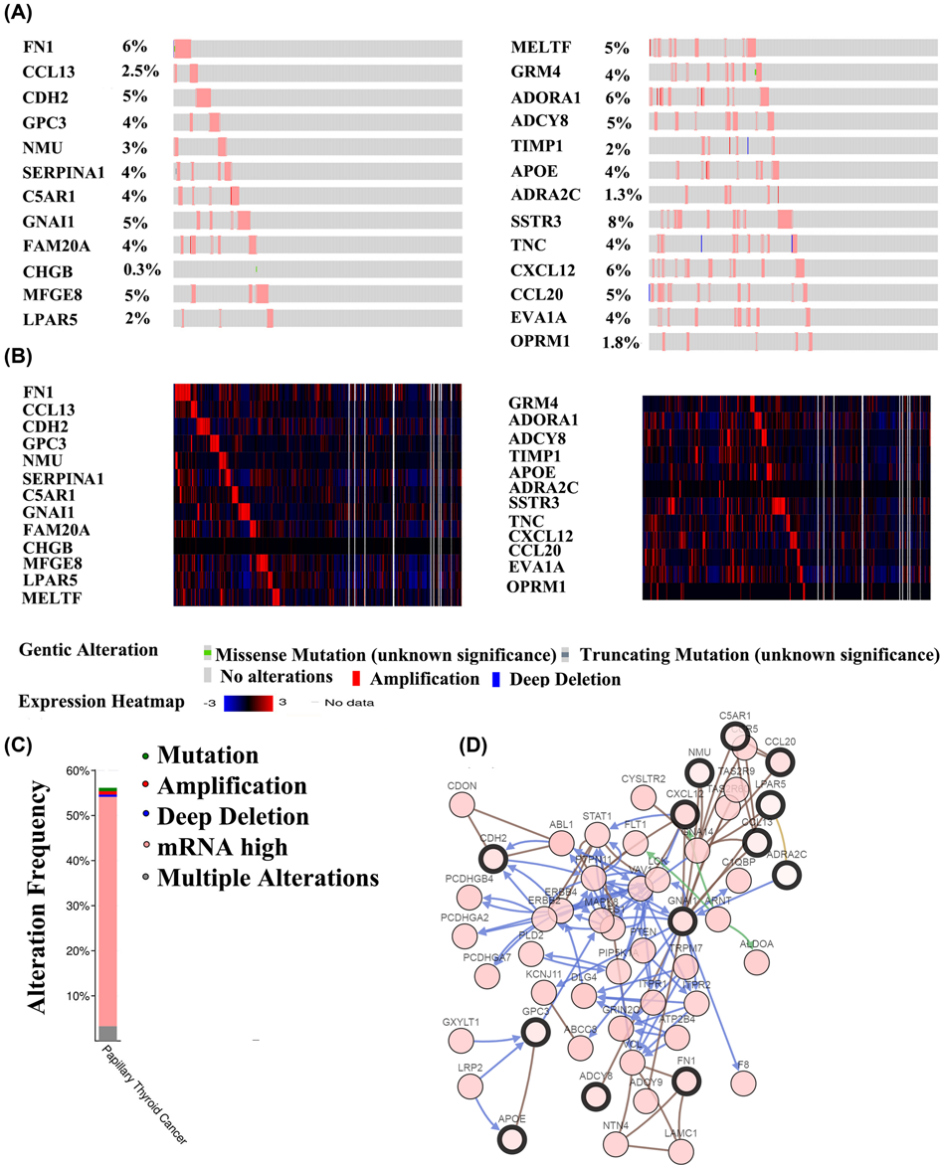
The 25 key genes expression and mutation analysis in PTC by the cBioPortal for Cancer Genomics (**A**) The genetic alterations of 25 key genes of 399 PTC samples. Queried genes are altered in 224 (56%) of queried patients/samples. (**B**) The expression heatmap of 25 key genes. (**C**) The alteration frequency of 25 key genes in PTC. (**D**) Network of 25 key genes mutations and their 50 frequently altered neighboring genes in PTC.

Next, we analyzed the alterations of the 25 key genes by using the cBioPortal database (Figure 7). The 25 key genes were changed in 224 (56%) of queried samples (Figure 7B). Figure 7A showed the frequency of alterations of each PTC related key gene. SSTR3, FN1, and ADORA1 were altered the most (8%, 6%, and 6%, respectively). Figure 7D showed the network of the 25 genes and their altered neighbouring genes in PTC patients (out of a total of 1278).

**Figure 8:**
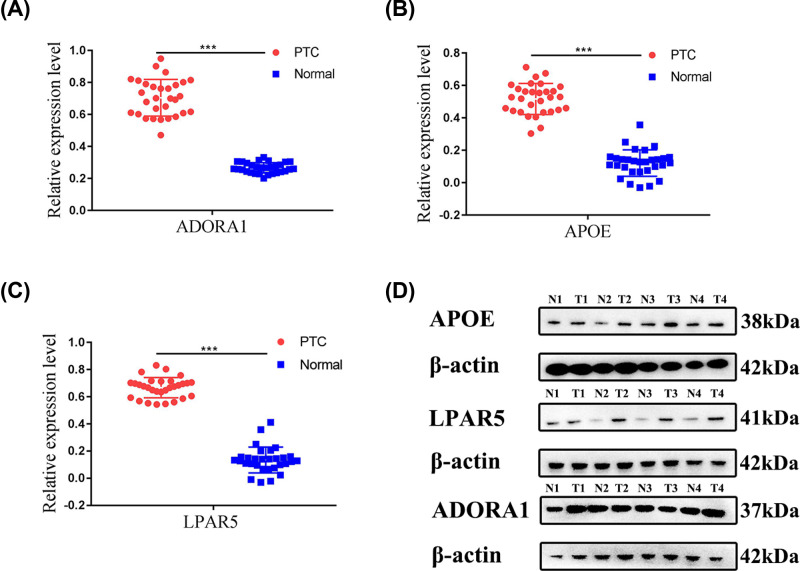
The mRNA and protein expressions of ADORA1, APOE, and LPAR5 in PTC tissues (**A–C**) Validation of expression levels of ADORA1, APOE, and LPAR5 by RT-qPCR in 30 cases of PTC and matched adjacent tissues. (**D**) ADORA1, APOE, and LPAR5 protein levels are increased in four cases of PTC and matched adjacent tissues, as measured by Western Blot; *** *P*<0.001.

**Figure 9:**
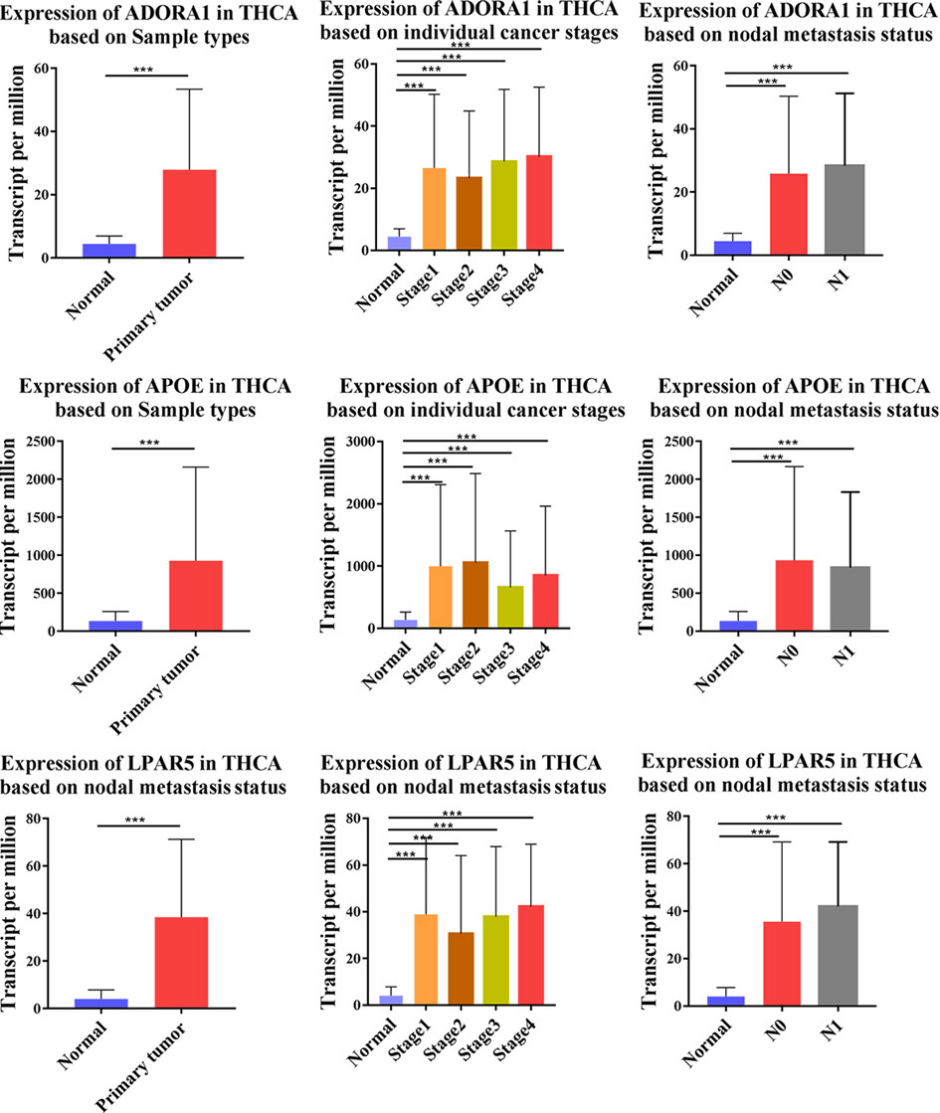
Relative expression of ADORA1, APOE, and LPAR5 in normal thyroid tissues and PTC tissues, individual cancer stages and nodal metastasis status, respectively (UALCAN) ***P<0.001

**Figure 10:**
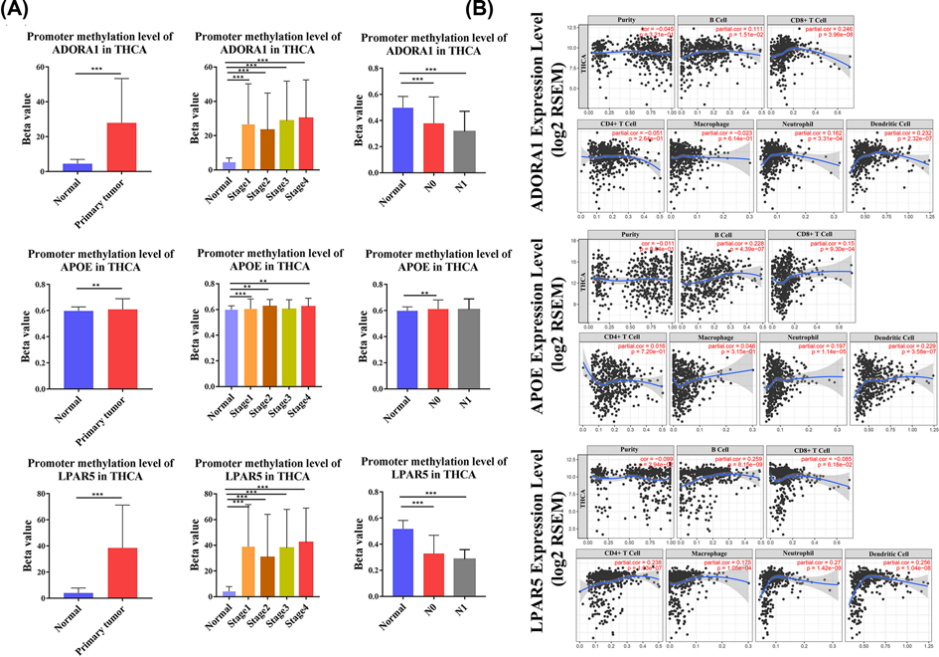
Methylation level and immune infiltration level of ADORA1, APOE, and LPAR5 (**A**) Relative methylation level of ADORA1, APOE and LPAR5 based on normal thyroid tissues and PTC tissues, individual cancer stages and nodal metastasis status, respectively (UALCAN). (**B**) The correlation between the three genes and TIICs (TIMER); TIICs, tumor infiltrating immune cells.

Among these genes, only ADORA1, APOE, and LPAR5 genes simultaneously showed statistical significance for overall survival analysis and disease-free survival analysis of PTC patients. The qPCR experiments and Western blot data verified that these three survival-related genes were all overexpressed in PTC (Figure 8). Then, based on UALCAN, the clinical features and degree of methylation of these three genes were analyzed. The transcription levels of ADORA1, APOE and LPAR5 were significantly higher in PTC patients than normal tissues according to subgroups of sample types, individual stages and nodal metastasis status (Figure 9). In addition, ADOR1 and LPAR5 exhibited a hypomethylation state in the cancer group, but APOE showed a hypermethylation state in PTC samples (Figure 10A).

**Figure 11:**
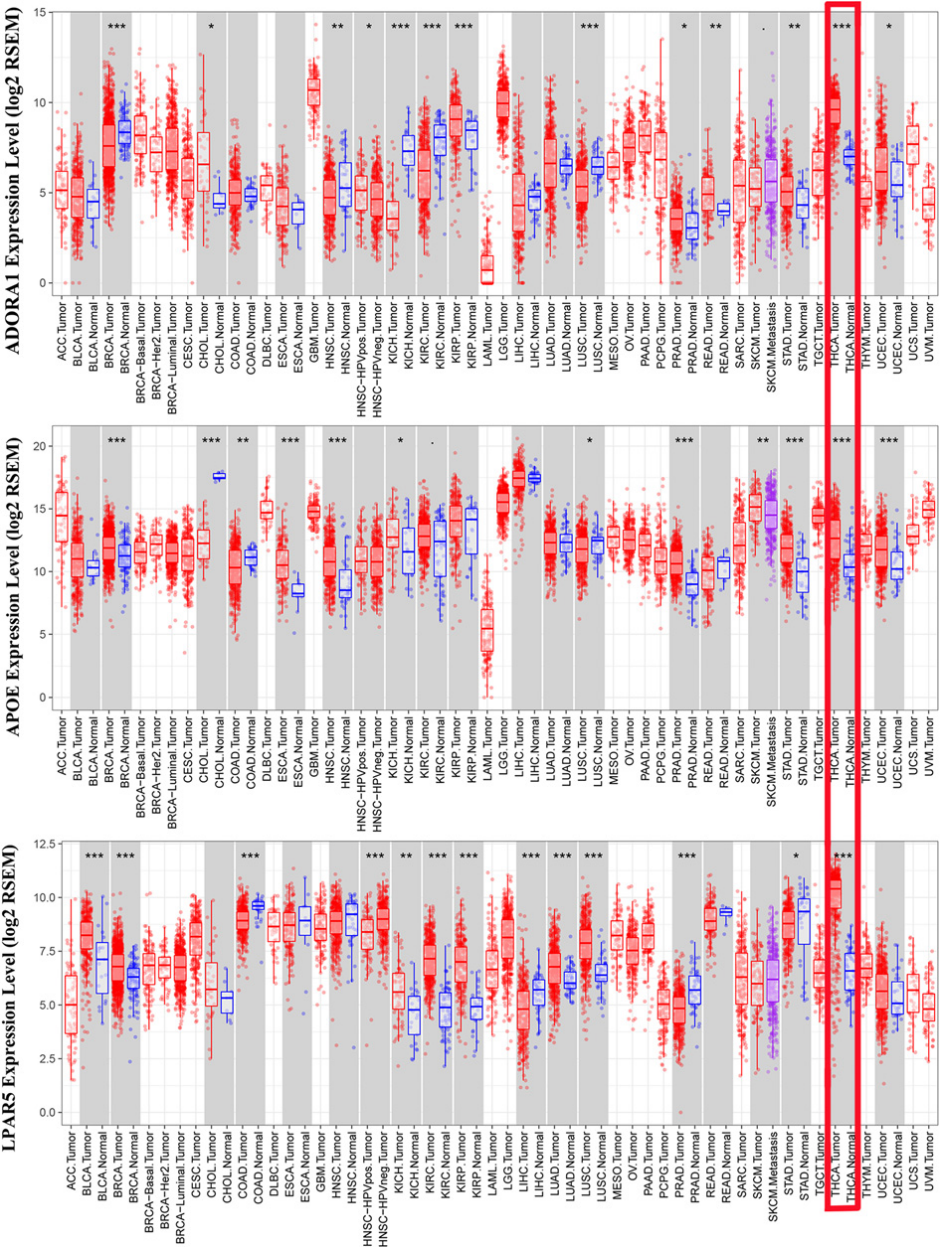
The expression of ADORA1, APOE, and LPAR5 in thyroid cancer tissues and normal thyroid tissues The three genes expression were analyzed in different kind of cancer tissues and normal tissues via the TIMER database; *P<0.05, **P<0.01, ***P<0.01.

To further clarify the role of these genes, we conducted an analysis of immune infiltration. The ADOR1 expression level was positively corelated with infiltrating levels of B cells (*r*=0.111, *P*=1.51e-2), CD8+ T cells (*r*=0.246, *P*=3.96e−08), neutrophils (*r*=0.162, *P*=3.31e−04) and DCs (*r*=0.232, *P*=2.32e−07). The expression of APOE was positively associated with B cells (*r*=0.228, *P*=4.39e−07), CD8+ T cells (partial.cor = 0.15, *P*=9.30e−04), neutrophils (*r*=0.197, *P*=1.14e−05), and DCs (partial.cor = 0.229, *P*=3.58e−07). LPAR5 expression level was positively related to B cells (*r*=0.259, *P*=8.15e−09), CD4+ T cells (*r*=0.238, *P*=1.03e−07), macrophages (*r*=0.175, *P*=1.05e−04), neutrophils (*r*=0.27, *P*=1.42e−09) and DCs (*r*=0.256, *P*=1.04e−08) and negatively related to Purity (*r*= −0.099, *P*=2.94e−02) and CD8+ T cells (*r* = −0.085, *P*=6.18e−02) (Figure 10B). These findings strongly suggested that LPAR5, ADOR1, and APOE may play specific roles in immune infiltration in PTC, especially those of DCs. Finally, we examined the expression of these three genes in common cancer tissues and adjacent tissues, and we found that these three genes were highly expressed in most cancer tissues (Figure 11).

## Discussion

PTC is a common cancer with great heterogeneity in morphological features and prognosis [12]. Although most papillary thyroid carcinomas exhibit low biological activity, there are still a few patients with higher invasive and metastatic clinical features [13]. Activation of oncogene expression and loss of function of tumor suppressor genes may lead to the development or progression of PTC. To better clarify the molecular mechanism of PTC occurrence, development and metastasis, we identified 25 key genes related to PTC progression through comprehensive bioinformatics methods, and we screened three of the PTC prognosis-related genes for a comprehensive analysis.

In the present study, we identified 2462 differentially expressed genes by RNA-Seq, with GO enrichment analysis showing that the DEGs were enriched in the regulation of the axonogenesis, regulation of cell morphogenesis, extracellular structure organization, extracellular matrix organization, synapse organization, cell–substrate adhesion, urogenital system development, collagen-containing extracellular matrix, synaptic membrane, cell–cell junction, glutamatergic synapse, neuron to neuron synapse, postsynaptic membrane, basolateral plasma membrane, cell adhesion molecule binding, passive transmembrane transporter activity, extracellular matrix structural constituent, glycosaminoglycan binding, growth factor binding, transmembrane receptor protein kinase activity, and transmembrane receptor protein tyrosine kinase activity. And KEGG pathway results showed DEGs were enriched in cytokine–cytokine receptor interaction, MAPK signaling pathway, proteoglycans in cancer, Rap1 signaling pathway, axon guidance, Cushing syndrome, parathyroid hormone synthesis, secretion and action, AGE-RAGE signaling pathway in diabetic complications, growth hormone synthesis, secretion and action, salivary secretion, circadian entrainment, cholinergic synapse, p53 signaling pathway, ECM–receptor interaction, arrhythmogenic right ventricular cardiomyopathy (ARVC), endocrine resistance, renin secretion, Type II diabetes mellitus, bladder cancer, nicotinate and nicotinamide metabolism, and apoptosis-multiple species.

To further explore the interrelationship of differentially expressed genes in papillary thyroid carcinoma, we constructed a PPI regulatory network. A total of 25 DEGs with nodes greater than 10 were screened out in the network. The key genes were ADCY8, ADORA1, ADRA2C, APOE, C5AR1, CCL13, CCL20, CDH2, CHGB, CXCL12, EVA1A, FAM20A, FN1, GNAI1, GPC3, GRM4, LPAR5, MELTF, MFGE8, NMU, OPRM1, SERPINA1, SSTR3, TIMP1, and TNC. Biological process and molecular function analyses of these 20 key DEGs indicated that they were significantly involved in cancer regulation processes such as adjustment of cell growth or maintenance, cell immune response, cell adhesion molecular activity, and extracellular matrix structural constituent.

To verify the credibility of the experiments and data, the 25 DEGs screened were verified by the GEPIA database. Among the 25 selected genes, 15 genes showed expression differences consistent with our RNA-Seq data. Among the 15 genes, ADORA1, APOE, CCL13, CDH2, EVA1A, FAM20A, FN1, LPAR5, MFGE8, NMU, SERPINA1, TIMP1, and TNC levels were overexpressed in PTC tissues while GPC3 and GNAI1 were down-regulated.

ADORA1 belongs to the G-protein coupled receptor 1 family and protects human tissues and cells under physiological conditions [14]. Lin et al. suggested that ADORA1 may promote the proliferation of breast cancer cells by positively regulating oestrogen receptor-alpha in breast cancer cells [15]. Similarly, Jayakar indicated that knockdown of APOE expression can reduce the level of MMPs by regulating the AP-1 signaling pathway and thus reduce the invasion and metastasis of oral squamous cell carcinoma [16]. Bioinformatics predictions were that APOE mRNA shows a significant increase in PTC [17]. CCL13 is a coding gene involved in immune regulation and inflammatory responses, and it has been reported that CCL13 has a role in promoting the proliferation of tumor-forming volume in nude mice [18]. CDH2 is overexpressed in various cancers. Some research results indicate that overexpression of CDH2 can increase the invasive ability and induce EMT in lung cancer cells [19]. Qiu et al. confirmed CDH2 acts as an oncogene in papillary thyroid carcinoma, which is consistent with our findings [20]. EVA1A acts as a regulator of programmed cell death, and Shen et al. indicates that EVA1A can inhibit the proliferation of GBM cells by inducing autophagy and apoptosis via inactivating the mTOR/RPS6KB1 signaling pathway [21]. FAM20A may play a key role in haematopoiesis. There are few reports on the relationship between FAM20A and cancer, and our experiment found that FAM20A is more highly expressed in papillary thyroid carcinoma than in other cancers. FN1 is involved in regulating cell adhesion, cell movement, wound healing, and maintaining cell morphology [22]. Some researchers indicated that FN1 participates in regulating many types of cancer progression, such as gastric cancer [23], skin squamous cell carcinoma [24], and papillary thyroid carcinoma [20,25,26]. It has been shown that LPAR5 is related to the pathogenesis of pancreatic cancer [27]. Consistent with our study, Zhang et al. believes that LPAR5 may be involved in the development of papillary thyroid carcinoma [28]. According to previous reports, MFG-E8 is involved in the progression of various malignancies, such as breast cancer, melanoma, bladder tumors, and ovarian cancer [29–32]. MFGE8 is considered to be a potential therapeutic target for ovarian cancer owing to its carcinogenic effect [32]. Consistent with our data, Zhang et al. indicate that NMU is one of the DEGs of papillary thyroid carcinoma [28]. Recently, a researcher has shown that abnormal expression of NMU is associated with a variety of cancers [33]. For SERPINA1, there are currently six articles pointing out that SERPINA1 may be a key gene for PTC, consistent with our results [28,34–38]. Clinical studies have shown that high expression of TIMP1 is positively correlated with a poor prognosis of colon, brain, prostate, breast, lung, and several other cancers [39]. TNC is a component of the extracellular matrix (ECM) and is closely related to the malignant biological behavior of cancer. In particular, TNC overexpression is positively associated with liver cancer, oral squamous cell carcinoma, and lymph node metastasis of breast cancer [40,41]. GPC3 belongs to the glypicans family. It has been reported that overexpression of GPC3 can promote the metastasis of hepatocellular carcinoma [42], but we found that it is expressed at low levels in PTC. Similar to GPC3, some scholars believe that GNAI1 is a tumor-promoting gene and reported up-regulated GNAI1 mRNA in human glioma, which is inconsistent with our data [43].

Only the ADORA1, APOE, and LPAR5 genes simultaneously showed statistical significance for overall survival and disease-free survival of PTC patients. Considering that the occurrence and metastasis of cancer is a complex and multi-regulated process, we further analyzed the regulatory mechanisms of these three genes. We found that the mRNA and methylation levels of these three genes were significantly correlated with TNM staging. In addition, ADORA1, APOE, and LPAR5 were all related to immune infiltration, especially to dendritic cells. Finally, we found that these three genes were more highly expressed in cancer tissues than matched adjacent tissues.

However, our research has certain limitations. First, only four pairs of cancer and adjacent tissues were analyzed using RNA-seq in this experiment, so further research requires a larger sample size. Second, further experiments are needed to validate the specific mechanisms of these key genes.

## Data Availability

The data used to support the findings of this study are available from the corresponding author upon request.

## Abbreviations

ATC: anaplastic thyroid carcinoma
ECM: extracellular matrix
FTC: follicular thyroid carcinoma
PTC: papillary thyroid carcinoma
